# Characterization of RT6-Bearing Rat Lymphocytes. II. Developmental Relationships of RT6^-^  and RT6^+^T Cells

**DOI:** 10.1155/1991/87067

**Published:** 1991

**Authors:** Christopher F. Mojcik, Dale L. Greiner, Irving Goldschneider

**Affiliations:** Department of Pathology School of Medicine University of Connecticut Health Center Farmington Connecticut 06030 USA

## Abstract

The derivation of RT6^+^ T cells from postthymic RT6^-^ T cells in weanling rats was formally demonstrated by the intravenous transfer (“parking”) of highly purified populations of RT6^-^ lymph node T cells into thymectomized, irradiated, and bone-marrow-reconstituted (TXBM) RT6^+^ and RT7 alloantigen-disparate recipients. Parallel experiments in irradiated and bonemarrow- reconstituted rats, and in rats whose RT6^+^ T cells had been depleted by injection of DS4.23 anti-RT6.1 mAb, suggested that the transit time between the pre-RT6^+^ and the RT6^+^ T-cell compartments approximated 4-5 days. A more precise estimate of the transit time was made by linear regression analysis of the generation of RT6^+^ T cells in rats that were treated with DS4.23 mAb at timed intervals after thymectomy. This study indicated that 50% of the pre-RT6 T cells differentiated into RT6^+^ cells within 4 days, 75% within 8 days, and more than 90% within 16 days.

Despite the apparent absence of pre-RT6^-^ T cells 3 weeks after thymectomy, numerous RT6^-^ T cells persisted for at least 10 weeks in thymectomized rats, even after treatment with DS4.23 mAb. Moreover, these RT6^+^ T cells failed to generate RT6^+^ T cells after transfer into adoptive hosts. Quantitative and phenotypic analyses indicated that this population of “true” RT6^-^ T cells: (1) constitutes approximately 50% of the total RT6^-^ T cells normally found in control rats; (2) contains CD4 and CD8 subsets; (3) expresses both the CD5 pan-T-cell antigen (which is absent from NK cells) and the R73 α/β TCR constant-region determinant; and (4) lacks sIgM.

Hence, the present results indicate that the “true” RT6^-^ and the RT6^+^ T-cell subsets have stable antigenic phenotypes and represent developmentally discrete populations of postthymic cells in normal rats. This is supported by associated phenotypic and functional studies that suggest that the “true” RT6^-^T-cell subset contains antigenically naive and/or autoreactive clonotypes, whereas the RT6^+^ T-cell subset contains memory and/or regulatory cells. It remains to be determined whether the “true” RT6^-^ and the RT6^+^ subsets represent separate lineages of T cells or a single lineage at different stages of activation or maturation.


*Developmental Immunology*, 1991, Vol. 1, pp. 191–201
Reprints available directly from the publisher
Photocopying permitted by license only

© 1991 Harwood Academic Publishers GmbH
Printed in the United Kingdom

### Characterization of RT6-Bearing Rat Lymphocytes. II. Developmental Relationships of RT6$^-$ and RT6$^+$ T Cells

CHRISTOPHER F. MOJCIK[†], DALE L. GREINER[†] and IRVING GOLDSCHNEIDER[*†]

[†]*Department of Pathology, School of Medicine, University of Connecticut Health Center, Farmington, Connecticut 06030*

The derivation of RT6$^+$ T cells from postthymic RT6$^-$ T cells in weanling rats was formally demonstrated by the intravenous transfer ("parking") of highly purified populations of RT6$^-$ lymph node T cells into thymectomized, irradiated, and bone-marrow-reconstituted (TXBM) RT6 and RT7 alloantigen-disparate recipients. Parallel experiments in irradiated and bone-marrow-reconstituted rats, and in rats whose RT6$^+$ T cells had been depleted by injection of DS4.23 anti-RT6.1 mAb, suggested that the transit time between the pre-RT6$^+$ and the RT6$^+$ T-cell compartments approximated 4–5 days. A more precise estimate of the transit time was made by linear regression analysis of the generation of RT6$^+$ T cells in rats that were treated with DS4.23 mAb at timed intervals after thymectomy. This study indicated that 50% of the pre-RT6$^+$ T cells differentiated into RT6$^+$ cells within 4 days, 75% within 8 days, and more than 90% within 16 days.

Despite the apparent absence of pre-RT6$^-$ T cells 3 weeks after thymectomy, numerous RT6$^-$ T cells persisted for at least 10 weeks in thymectomized rats, even after treatment with DS4.23 mAb. Moreover, these RT6$^+$ T cells failed to generate RT6$^+$ T cells after transfer into adoptive hosts. Quantitative and phenotypic analyses indicated that this population of "true" RT6$^-$ T cells: (1) constitutes approximately 50% of the total RT6$^-$ T cells normally found in control rats; (2) contains CD4$^+$ and CD8$^+$ subsets; (3) expresses both the CD5 pan-T-cell antigen (which is absent from NK cells) and the R73 $\alpha/\beta$ TCR constant-region determinant; and (4) lacks sIgM.

Hence, the present results indicate that the "true" RT6$^-$ and the RT6$^+$ T-cell subsets have stable antigenic phenotypes and represent developmentally discrete populations of postthymic cells in normal rats. This is supported by associated phenotypic and functional studies that suggest that the "true" RT6$^-$ T-cell subset contains antigenically naive and/or autoreactive clonotypes, whereas the RT6$^+$ T-cell subset contains memory and/or regulatory cells. It remains to be determined whether the "true" RT6$^-$ and the RT6$^+$ subsets represent separate lineages of T cells or a single lineage at different stages of activation or maturation.

KEYWORDS: Rat T-cell development, T-cell subsets, RT6 and RT7 alloantigens, differentiation antigens, monoclonal antibodies.

### INTRODUCTION

RT6 is a low-molecular-weight cell-surface protein that exists in two known allelic forms and is displayed exclusively by members of the T-lymphocyte lineage in the rat (Lubaroff et al., 1979; Greiner et al., 1982; Thiele et al., 1987; Mojcik et al., 1988). Both the RT6.1 and RT6.2 alloantigens are attached to the cell-surface membrane by phosphatidylinositol linkages (Koch et al., 1986; Koch et al., 1988), however, the RT6.1 alloantigen is glycosylated, whereas the RT6.2 alloantigen is not.

*Corresponding author.

Approximately 70% of peripheral T cells, including both CD4$^+$ and CD8$^+$ subsets, express RT6, but no RT6$^+$ thymocytes have been detected (Mojcik et al., 1988). Some RT6$^+$ T cells, their precursors, and/or their progeny respond to mitogenic and allogeneic stimulation *in vitro*, secrete interleukin-2, and participate in the graft-vs.-host (Greiner et al., 1982; Hunt and Lubaroff, 1985) and delayed-type hypersensitivity reactions *in vivo* (Ernst and Lubaroff, 1984). Furthermore, RT6$^+$ T cells have been shown to play an important regulatory role in suppressing mixed lymphocyte reactivity *in vitro* (Hunt and Lubaroff, 1985) and in preventing autoimmunity in the spontaneously hyperglycemic BB/

Wor rat (Greiner et al., 1987), as well as in normal rats (McKeever et al., 1990). In contrast, at least some RT6⁻ T cells may serve as autoreactive effector cells in the absence of RT6⁺ T cells (Greiner et al., 1986; McKeever et al., 1990).

Previously, we have shown that the appearance of RT6⁺ T cells occurs later in ontogeny than does that of RT6⁻ T cells, and does not reach maximal levels until 6–8 weeks of age (Mojcik et al., 1988). Intrathymic adoptive transfer studies demonstrated that most, if not all, RT6⁺ T cells are thymus-derived; and neonatal thymectomy experiments provided suggestive evidence for a parent–progeny relationship between RT6⁻ and RT6⁺ T cells. Definitive evidence for such a relationship is provided in the present study using purified subsets of RT6⁻ and RT6⁺ T cells. These RT6⁻ precursors of RT6⁺ cells are referred to as "pre-RT6⁺" T cells. In addition, our results suggest that another population of RT6⁻ T cells exists that does not contain the precursors of RT6⁺ T cells. We have termed these lymphocytes "true RT6⁻" T cells, and have postulated that they may represent a developmentally discrete population of postthymic lymphocytes with unique functional attributes.

### RESULTS

### Parent–Progeny Relationship of RT6⁻ and RT6⁺ T Cells

*a. Adoptive transfer studies.* To investigate formally the developmental relationships of RT6⁻ and RT6⁺ T-cell subsets, purified populations of RT6⁻ and RT6⁺ lymph-node T cells from BUF rats were injected *i.v.* into histocompatible TXBM M520 recipients. Donor-origin RT6⁻ and RT6⁺ T cells in spleen and lymph node were quantified 6 weeks later by double immunofluorescence analysis for the RT6.1 (T-cell subset) and RT7.2 (pan-T-cell) alloantigens.

As shown in Table 1, normal percentages (approximately 70%) of RT6⁺ donor-origin T cells were observed in adoptive recipients of either unfractionated lymph-node T cells or purified (>98%) RT6⁻ T cells obtained from nonthymectomized rats. In contrast, when purified populations of RT6⁺ T cells were adoptively transferred, only RT6⁺ donor-origin T cells were detected in the recipients. As anticipated (Greiner et al., 1982; Mojcik et al., 1988), no donor-origin RT6⁺ or RT6⁻ T cells whatsoever appeared in TXBM recipients of BUF bone marrow cells (data not shown).

Conversion of the results to whole-body T cells (Rossini et al., 1986) by the method of Everett et al. (1964), showed that at 6 weeks after cell transfer, the total number of donor-origin T cells in the recipients of either unfractionated, purified RT6⁻, or purified RT6⁺ T cells closely approximated the number of T cells in the original inoculum (Table 1). Moreover, $16.8 \times 10^6$ (84%) of the $20 \times 10^6$ donor-origin T cells present in the recipients of purified RT6⁻ T cells were RT6⁺.

*b. Transit time between the pre-RT6 and RT6⁺ T-Cell compartments.* Previous ontogenetic studies had suggested that 4 days was the minimal interval required between the release of RT6⁻ T cells from the thymus and the expression of the RT6 antigen on their surface (Mojcik et al., 1988). To gain further information about the kinetics of differentiation, bone marrow cells from young adult BUF rats were injected *i.v.* into irradiated (750 rad) M520 recipients and the appearance of donor-origin (RT7.2⁺) RT6⁺ and RT6⁻ T cells was determined. As shown in Fig. 1, donor-origin RT6⁻ T cells were first detected in both spleen and lymph node on day 25, whereas donor-origin RT6⁺ T cells were first detected in these tissues on day 30. Total and RT6⁺ donor-origin T cells increased rapidly and in parallel thereafter, and began to plateau at 6–8 weeks. As in normal ontogeny (Mojcik et al., 1988), the relative proportion of T cells that expressed RT6 increased progressively during the second month after bone-marrow-cell transfer, and reached stable adult levels (approximately 70%) within 3 months. In addition, both OX8⁺ (CD8) and W3/25⁺ (CD4) subsets of RT6⁺ T cells were generated with kinetics similar to those observed during normal ontogeny (Mojcik et al., 1988; data not shown).

In a second series of experiments in young adult LEW rats, RT6⁺ T cells were depleted (>99%) by a single *i.v.* injection of DS4.23 (anti-RT6.1) mAb (see Materials and Methods). As shown in Table 2, although many T cells (RT7⁺) remained in spleen and lymph node at 24 hr, RT6⁺ T cells were not detected until day 5 after DS4.24 treatment. Thereafter, the percentage of total T cells that expressed RT6 increased progressively to reach approximately 70% of control levels in spleen and lymph node by day 21.

To control for the possible effects that the continued release of RT6⁻ T cells from the thymus may have had on the pool of pre-RT6⁺ T cells and/or their rate of differentiation into RT6⁺ T cells, young

TABLE 1

Ability of $RT6^+$ and $RT6^-$ T-Cell Subsets from BUF Rat Lymph Node to Repopulate the T-Cell Compartments of TXBM M520 Rats[a]

| BUF T-cell innoculum | | | | Recovery of donor-origin T cells in M520 recipients | | | | | | |
|---|---|---|---|---|---|---|---|---|---|---|
| | | | | Donor $RT7.2^+$ T cells | | | Donor $RT6.1^+$ T cells | | | |
| T-cell subset | No. cells injected ($\times 10^{-6}$) | % $RT6^+$ T cells | Organ | % in organ | Total in organ ($\times 10^{-6}$) | Total in body[b] ($\times 10^{-6}$) | % in organ | Total in organ ($\times 10^{-6}$) | Total in body[b] | % $RT6^+$/$RT7^+$ |
| Total T cells | 23 | 52.4 | LN | 14.4 ± 1.7 | ND | ND | 10.0 ± 2.7 | ND | ND | 69.4 |
| | | | Spl | 8.4 ± 2.7 | 9.2 | 23.0 | 5.8 ± 3.4 | 6.3 | 15.6 | 69.1 |
| Total $RT6^+$ | 25 | 94.5 | LN | 5.6 ± 3.7 | ND | ND | 5.6 ± 3.7 | ND | ND | 100.0 |
| | | | Spl | 4.4 ± 1.4 | 8.4 | 21.0 | 4.4 ± 1.4 | 8.4 | 21.0 | 100.0 |
| Total $RT6^-$ | 22 | 2.0 | LN | 11.1 ± 7.8 | ND | ND | 7.2 ± 4.2 | ND | ND | 64.9 |
| | | | Spl | 6.0 ± 2.2 | 8.0 | 20.0 | 5.0 ± 1.8 | 6.7 | 16.8 | 83.3 |
| "True" $RT6^-$ | 24 | 1.3 | LN | 8.4 | ND | ND | 0.4 | ND | ND | 4.8 |
| | | | Spl | 3.6 ± 0.3 | 4.0 | 10.0 | 0.4 ± 1.3 | 0.4 | 1.0 | 10.0 |

[a]Thymectomized, irradiated, and syngeneic bone-marrow-reconstituted (TXBM) adult M520 (RT6.2, RT7.1) rats were injected with one of the following BUF (RT6.1, RT7.2) rat lymph-node T-cell populations: Total (unfractionated) T cells enriched by nylon wool passage; $RT6^+$ T cells enriched by "panning" using $F(Ab')_2$ DS4.23 (anti-RT6.1) mAb; total $RT6^-$ T cells obtained 24 hr after treatment of normal rats with intact DS4.23 (anti-RT6.1) mAb; or "true" $RT6^-$ T cells obtained from rats thymectomized 4 weeks before treatment with intact DS4.23 mAb (see Materials and Methods). Six weeks after adoptive transfer, recipients were analyzed for donor origin RT7.2 and RT6.1 T cells by flow cytofluorimetry. Results represent the mean ± S.D. of three to four animals per group or were obtained from pooled tissues of three to four animals. ND: not determined.

[b]Total donor-origin $RT7^+$ and $RT6^+$ cells in the body were calculated by the method of Everett et al. (1964), assuming spleen contains 40% of total body T lymphocytes.

adult LEW rats were injected with DS4.23 mAb at different times after thymectomy. These rats were then analyzed for $RT6^+$ T cells 2 weeks after cessation of mAb treatment. In order to be certain that all $RT6^+$ T cells were destroyed, DS4.23 mAb was administered on two alternate days.

Results in Table 3 show that injection of DS4.23 mAb between days 3 and 15 after thymectomy cause a progressive decrease in the number of spleen cells that became $RT6^+$; and no $RT6^+$ T cells were generated within 2 weeks by rats that received DS4.23 mAb on day 21 after thymectomy. Moreover, regression analysis of the decrease in the number of

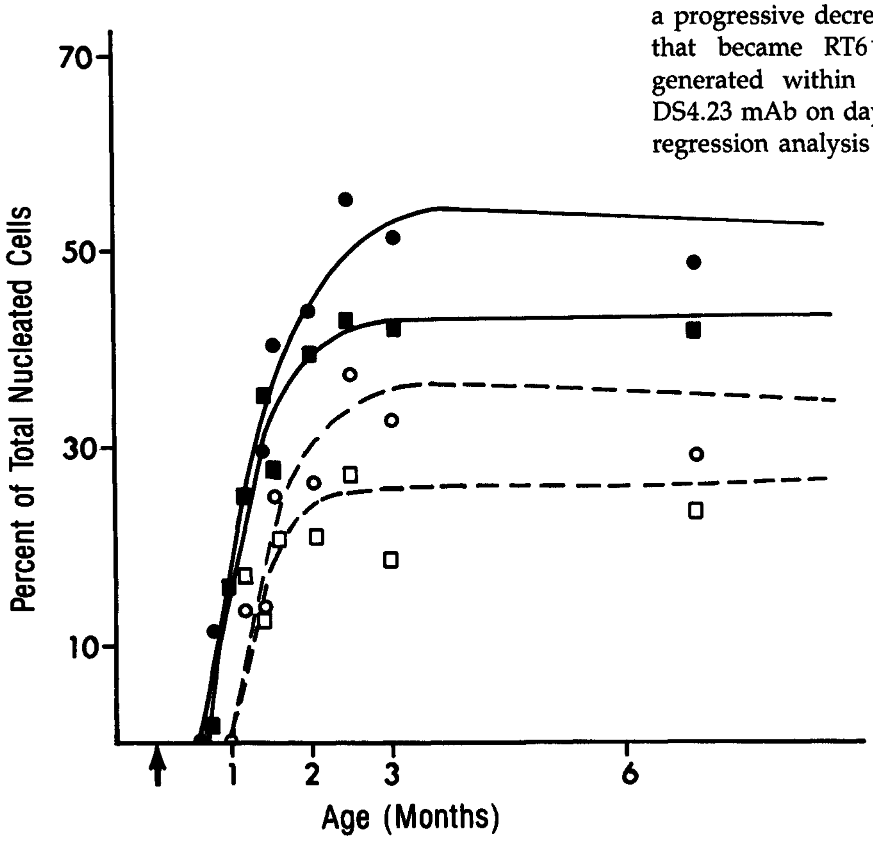

FIGURE 1. Kinetics of appearance of donor-origin $RT7^+$ (total) (solid line) or $RT6^+$ (dashed line) T cells in lymph node (●, ○) and spleen (■, □) of histocompatible, RT6- and RT7-disparate M520 rats with time after 750 R irradiation and adoptive transfer of BUF bone marrow cells *i.v.* Each point represents the mean of three to six recipients, as determined by flow cytofluorimetry.

TABLE 2
Kinetics of Reappearance of RT6.1⁺ T Cells in Nonthymectomized LEW Rats Following Injection of DS4.23 (anti-RT6.1) mAb[a]

| Days after injection of DS4.23 mAb | Percentage of positive cells | | | | | |
|---|---|---|---|---|---|---|
| | Spleen | | | Lymph node | | |
| | $RT6.1^+$ | $RT7.1^+$ | $RT6^+/RT7^+$ | $RT6.1^+$ | $RT7.1^+$ | $RT6^+/RT7^+$ |
| 1 | <1 | 39.9 ± 0.6[b] | 0.0 | <1 | 50.9 ± 2.9[b] | 0.0 |
| 3 | <1 | ND[c] | ND | <1 | ND | ND |
| 5 | 8.9 | ND | ND | 4.7 | ND | ND |
| 7 | 14.4 ± 1.9 | 38.9 ± 1.9 | 37.0 | 23.5 ± 3.0 | 54.9 ± 3.9 | 42.8 |
| 21 | 22.3 ± 2.4 | 46.2 ± 2.5 | 48.3 | 27.1 ± 6.5 | 56.7 ± 6.4 | 47.8 |
| Control | 34.7 ± 3.7 | 60.1 ± 6.1 | 57.7 | 46.9 ± 1.3 | 70.2 ± 2.8 | 66.8 |

[a]Each rat received a single *i.v.* injection of 0.3 mg/kg DS4.23 (anti-RT6.1) mAb. Results represent the mean ± S.D. of three to six animals per group, as determined by flow cytofluorimetry. Cells from day 5 were pooled from four rats prior to analysis. Control is age-matched for the day 21 time point.
[b]The total number of $RT7.1^+$ T cells was decreased by 59.2% in spleen and 67.6% in lymph node.
[c]ND: not determined.

$RT6^+$ cells as a function of increasing time between thymectomy and the administration of DS4.23 mAb (Fig. 2) shows that: (1) it was strictly linear ($r^2$=0.99) through day 12; (2) 50% of the pre-RT6 cells passed from the $RT6^-$- to the $RT6^+$-cell compartment in approximately 4 days; and (3) the baseline (no detectable $RT6^+$ cells) was intercepted at day 16.

TABLE 3
Kinetics of Reappearance of RT6.1⁺ T cells in the Spleen of LEW Rats Following the Injection of Anti-RT6.1 (DS4.23) mAb at Various Times after Thymectomy[a]

| Days after thymectomy on which DS4.23 mAb was injected | Number of $RT6^+$ cells in spleen 2 weeks later[b] ($\times 10^{-6}$) |
|---|---|
| No Antibody | 16.0 ± 3.2 |
| 1 and 3 | 10.1 ± 4.5 |
| 4 and 6 | 5.2 ± 2.9 |
| 7 and 9 | 3.7 ± 0.6 |
| 10 and 12 | 1.8 ± 0.6 |
| 13 and 15 | <1 |
| 16 and 18 | <1 |
| 19 and 21 | <1 |

[a]Weanling LEW rats were thymectomized and injected with DS4.23 mAb (anti-RT6.1) mAb (0.3 mg/kg body weight) on the indicated days following thymectomy. Rats were sacrificed 2 weeks after the second injection. Results represent mean ± S.D. of three to four animals per group, as determined by flow cytofluorimetry.
[b]See Fig. 2 for the linear regression analysis of data.

### Evidence for a Separate Population of "True" $RT6^-$ T Cells

The results of the preceding study indicated that rats thymectomized approximately 2.5 weeks previously lack pre-$RT6^+$ T cells. Yet previous observations (Mojcik et al., 1988) demonstrated that substantial numbers of splenic $RT6^-$ T cells remained 6 and 10 weeks after thymectomy. This suggested that not all $RT6^-$ T cells are pre-$RT6^+$ T cells.

To test this hypothesis, young adult LEW rats were thymectomized, treated with DS4.23 mAb 4 weeks later, and assayed for $RT6^+$ T cells in the spleen 6 weeks thereafter. Results in Table 4 show that only 8.1% of the total T cells were $RT6^+$ in the thymectomized, DS4.23-injected animals, whereas approximately 59%, 67%, and 40% of the T cells were $RT6^+$ in control rats that were sham-thymectomized, thymectomized only, or DS4.23-injected only, respectively. Yet the thymectomized, DS4.23-injected rats contained approximately 62% and 50% of the total number of $RT6^-$ T cells as did the sham-thymectomized and DS4.23-injected control rats. Moreover, despite the major difference in the number of $RT6^+$ T cells ($1.5 \times 10^6$ vs. $21.6 \times 10^6$) in the thymectomized, DS4.23-injected and the thymectomized, non-DS4.23-injected rats, there was no difference between the number of $RT6^-$ T cells in these two groups of rats ($15.8 \times 10^6$ vs. $10.9 \times 10^6$).

Similar results were obtained in a group of LEW rats that were treated with DS4.23 mAb 4 weeks after thymectomy and assayed for $RT6^+$ spleen cells 3 weeks (instead of 6 weeks) later. Only 4.1% ($0.5 \times 10^6$) of the $RT7^+$ T cells were $RT6^+$ in the thymectomized, DS4.23-injected rats, as compared to 48.2% ($23.9 \times 10^6$) in the sham-thymectomized, DS4.23-injected control rats. Again the thymectomized, DS4.23-injected rats contained approximately 50% of the total number of $RT6^-$ T cells as did the sham-thymectomized, DS4.23-injected controls (data not shown).

To demonstrate further that not all $RT6^-$ T cells are the precursors of $RT6^+$ T cells, purified populations of RT6⁻ lymph-node T cells, obtained from BUF rats thymectomized 4 weeks prior to treatment with DS4.23 mAb, were adoptively transferred into TXBM M520 recipients. Results in Table 1 (bottom line) show that less than 10% of the donor-origin T cells expressed RT6 antigen 6 weeks later, and that the total number of RT6⁻ T cells that remained was approximately half of the number that was originally transferred. This is in marked contrast to results obtained after the transfer of purified RT6⁻ T cells from nonthymectomized donors, in which 83% of the donor-origin T cells expressed RT6 antigen 6 weeks later and the total number of T cells recovered approximated the total number of T cells transferred.

### Antigenic Phenotypes of "True" RT6⁻ T Cells

The antigenic phenotypes of the lymphoid cells in spleen and lymph node of LEW rats was determined 4 weeks after thymectomy. T cells were identified by the expression of the RT7.1 alloantigen and the R73 TCR framework antigen; B cells by the expression of sIg. Results in Table 5 show that the percentage of T cells decreased moderately in the thymectomized rats as compared to sham-thymectomized controls, and that this decrease was accompanied by a compensatory increase in the percentage of sIg⁺ B cells. In addition, the percentage of RT6⁺ T cells and the CD4 (W3/25):CD8 (OX8) ratios (2.6:1) in the thymectomized rats were not significantly different than control values.

In contrast, the percentage of T cells was markedly decreased in rats that were injected with DS4.23 (anti-RT6.1) mAb 4 weeks after thymectomy; and the percentage of RT6⁺ cells was reduced from 29% to 0% and from 49% to 1% in spleen and lymph node, respectively. Nonetheless, the CD4:CD8 ratio was normal or slightly elevated in the thymectomized (and sham-thymectomized), DS4.23-treated rats; and, as shown in Table 6, normal percentages of T cells expressed the OX19 (CD5) and R73 ($\alpha/\beta$ TCR) antigens.

Lastly, the results of Table 7 show that the number of total (RT7⁺) T cells decreased 42% and the number of RT6⁺ and RT6⁻ T cells decreased 35 and 50%, respectively, in the spleens from the rats thymectomized 4 weeks previously. In contrast, the number of total T cells decreased 73% in the rats that were injected with DS4.23 mAb 4 weeks after thymectomy, reflecting the complete elimination of residual RT6⁺ T cells. As anticipated, the number of RT6⁻ T cells in both thymectomized and sham-thymectomized rats was unaffected by treatment with DS4.23 mAb; and the number of splenic B cells was unaffected by either thymectomy and/or DS4.23 mAb treatment.

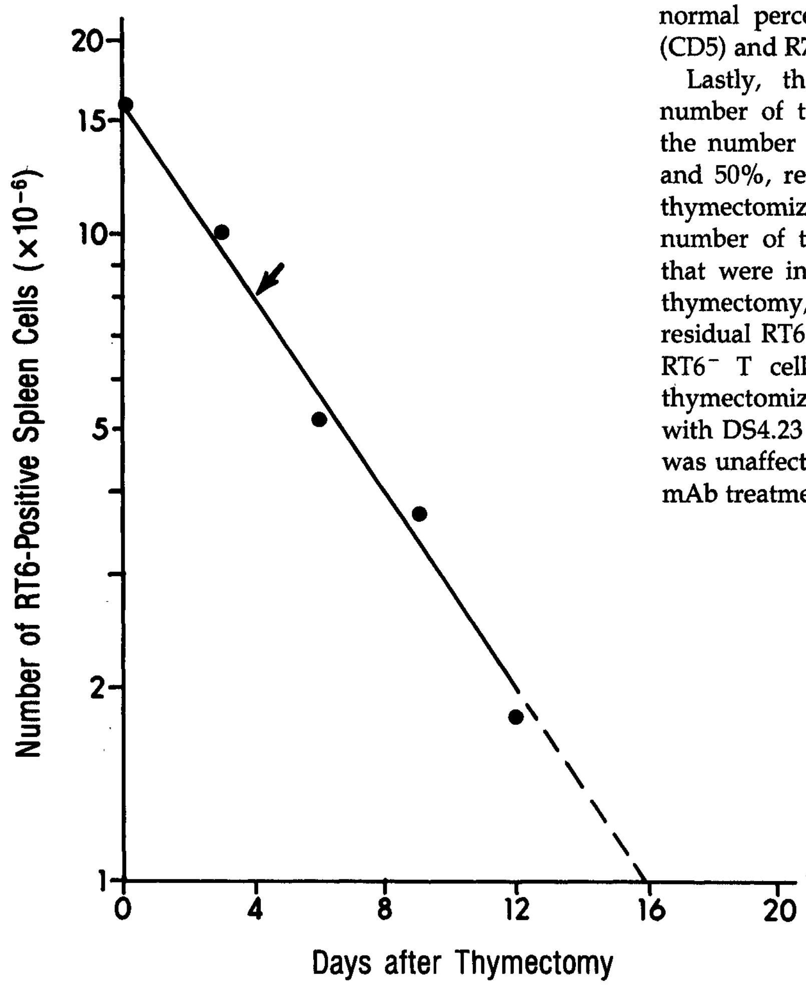

FIGURE 2. Linear regression analysis ($r^2$=0.99) of the reappearance of RT6.1⁺ T cells in the spleen of LEW rats 2 weeks after their depletion by the injection of DS4.23 (anti-RT6.1) mAb at indicated times after thymectomy (see footnote from Table 3 for details). Intercept of ordinate indicates number of RT6⁺ T cells in thymectomized but non-DS4.23-treated control rats. Intercept at abscissa (dashed line) indicates projected day after thymectomy after which no detectable RT6⁺ T cells are regenerated. Arrow designates approximate time after thymectomy during which 50% of the total RT6⁺ T cells are regenerated by RT6⁻ precursors.

TABLE 4
Frequency of RT6+ T Cells in Spleen of Thymectomized LEW Rats Six Weeks after Treatment with Anti-RT6.1 (DS4.23) mAb[a]

| Treatment of rats | | % Positive T cells | | | No. positive T cells ($\times10^{-6}$) | | |
|---|---|---|---|---|---|---|---|
| Tx | DS4.23 | RT7+ | RT6+ | RT6+/RT7+ | RT7+ | RT6+ | RT6− |
| − | − | 31.6±6.1 | 23.3±2.9 | 59.2 | 68.1±4.4 | 47.4±13.4 | 25.6±8.2 |
| + | − | 25.5±6.4 | 17.2±1.7 | 67.5 | 31.0±9.7 | 21.6±8.8 | 10.9±2.9 |
| − | + | 37.2±1.3 | 14.7±1.6 | 39.5 | 53.8±7.0 | 21.7±2.1 | 32.1±7.1 |
| + | + | 21.1±4.2 | 1.7±1.1 | 8.1 | 17.3±6.5 | 1.5±1.0 | 15.8±5.8 |

[a]Weanling LEW rats were thymectomized, treated with 0.3 mg/kg DS4.23 mAb 4 weeks later, and sacrificed 6 weeks thereafter (10 weeks total elasped time since thymectomy). Age-matched control rats were either sham-thymectomized and injected with buffer, thymectomized only, or treated with DS4.23 mAb only. Harvested nucleated spleen cells were analyzed for RT7.1 and RT6.1 antigens by flow microfluorimetry. Results represent the mean ±S.D. of four to six animals per group.

TABLE 5
Antigenic Phenotypes of Lymphocytes in Thymectomized and/or Anti-RT6.1 (DS4.23) mAb-Treated LEW Rats[a]

| Treatment of rats | | | % Positive lymphocytes | | | | | | |
|---|---|---|---|---|---|---|---|---|---|
| Tx | DS4.23 | Tissue | R73 | RT7.1 | RT6.1 | RT6.1/RT7.1 | W3/25 | OX8 | sIg |
| − | − | Spl | 66.2±2.3 | 56.2±0.5 | 29.1±0.8 | 51.8 | 50.6±0.7 | 21,8±1.4 | 33.4±0.8 |
| + | − | Spl | 49.9±2.7 | 45.0±2.2 | 26.3±1.3 | 58.4 | 43.7±0.7 | 16.8±1.5 | 40.8±2.0 |
| − | + | Spl | ND | 40.3±2.3 | 0.0±0.0 | 0.0 | 38.0±4.1 | 15.3±1.7 | 49.3±2.1 |
| + | + | Spl | 18.7±2.1 | 23.0±2.8 | 0.0±0.0 | 0.0 | 24.0±1.6 | 10.1±0.8 | 60.2±2.5 |
| − | − | LN | | 68.3±2.7 | 48.9±2.7 | 71.6 | 58.2±2.9 | 22.3±0.7 | 26.3±3.0 |
| + | − | LN | | 44.7±0.8 | 36.2±1.4 | 81.0 | 43.3±3.4 | 17.0±1.2 | 49.1±0.6 |
| − | + | LN | | 42.8±9.0 | 1.6±1.5 | 3.7 | 46.6±2.3 | 12.2±2.2 | 57.4±0.9 |
| + | + | LN | | 19.4±3.7 | 1.0±0.6 | 5.2 | 24.0±1.9 | 6.2±1.0 | 68.2±5.1 |

[a]Weanling LEW rats were thymectomized, treated with 0.3 mg/kg DS4.23 mAb 4 weeks later, and sacrificed 24 hr thereafter. Age-matched control rats were either sham-thymectomized and injected with buffer, thymectomized only, or treated with DS4.23 mAb only. The antigenic phenotypes of the nucleated spleen (Spl) and lymph-node (LN) cells were determined by flow microfluorimetry. Results represent the mean±S.D. of three animals per group. ND: not determined.

### DISCUSSION

In a previous report (Mojcik et al., 1988), we demonstrated that the expression of the RT6 alloantigen on a subset of T cells is a postthymic maturational event, and we provided indirect evidence that RT6+ T cells are derived from RT6− T cells (i.e., "pre-RT6+" T cells). We also suggested that a second population of RT6− T cells may exist, one that does not generate RT6+ T cells (i.e., "true RT6−" T cells). The results of the present study strongly support the existence of both pre-RT6+ and true RT6− T-cell subsets.

Adoptive transfer ("parking") experiments in TXBM rats showed that normal proportions of RT6+ T cells are generated within 6 weeks by purified populations of RT6− T cells obtained from nonthymectomized donors, and that the expression of the RT6 antigen is not dependent upon the continued presence of a thymus. The demonstration that TXBM recipients of purified RT6+ T cells con-

TABLE 6
Expression of OX19 (CD5) and RT7 (TCR) Antigens by T Lymphocytes in Spleen of Thymectomized (TX) and/or Anti-RT6.1 (DS4.23) mAb-Treated LEW Rats[a]

| Treatment of rats | | % of T lymphocytes (RT7+) that express | |
|---|---|---|---|
| Tx | DS4.23 | OX19 | R73 |
| − | − | 78.4±3.8 | 87.0±2.1 |
| + | − | 77.6±3.4 | 83.9±1.2 |
| − | + | 78.1±2.6 | ND |
| + | + | 71.7±2.4 | 70.6±7.7 |

[a]See footnote from Table 5 for treatment of rats. The percentage of doubly labeled cells was determined by two-color fluorescence microscopy. Results represent the mean±S.D. of three animals per group. ND: not determined.

tained only RT6$^+$ T cells 6 weeks later strongly suggests that the differentiation of RT6$^+$ T cells from RT6$^-$ precursors is unidirectional. This does not exclude the possibility, however, that activated RT6$^+$ T cells might transiently fail to express the RT6 antigen, as suggested by Hunt and Lubaroff (1987), or that some RT6$^+$ T cells might themselves generate additional RT6$^+$ T cells.

Studies of the sequential appearance of RT6$^-$ and RT6$^+$ T cells in irradiated and BM-reconstituted adult rats and in rats whose RT6$^+$ T cells had been ablated by treatment with the DS4.23 mAb suggested that the minimum transit time between the pre-RT6$^+$ and RT6$^+$ T-cell compartments was 4–5 days. Similar kinetics have been observed during normal ontogeny (Mojcik et al., 1988). However, given the continued contribution of the thymus to the peripheral T-cell pool in the preceding experiments, other explanations for the delayed appearance of RT6$^+$ cells are possible, such as the existence of separate precursor-cell populations for RT6$^-$ and RT6$^+$ T cells, each having different generative kinetics. Therefore, it is significant that experiments in thymectomized and DS4.23 mAb-treated adult rats not only confirmed the presence of pre-RT6$^+$ T cells, but identified a developmentally unrelated (or more primitive) population of true RT6$^-$ T cells.

In these latter experiments, the population of pre-RT6$^+$ T cells decreased exponentially with time after thymectomy, such that 50% of the precursor activity was absent by four days, 90% by 12 days, and virtually all by 21 days (see Table 3). Yet, despite the apparent absence of pre-RT6$^+$ cells, approximately 50% of the original number of RT6$^-$ T cells were present 4 weeks after thymectomy (see Table 7). Similarly, at 10 weeks, the number of RT6$^-$ T cells that were recovered from the spleens of thymectomized and DS4.23 mAb-treated rats was approximately 50% of that recovered from nonthymectomized, DS4.23-treated controls (see Table 4). Equivalent results were obtained by comparing the residual numbers of donor-origin RT6$^-$ T cells in TXBM recipients of purified RT6$^-$ T cells from normal and thymectomized rats (see Table 1). Hence, it appears that, under steady-state conditions in nonthymectomized rats, approximately half of the RT6$^-$ T cells are true RT6$^-$ T cells.

Although it is possible that some RT6$^-$ T cells may require more than 4 weeks to express the RT6 antigen, it is unlikely that this applies to the vast majority of the RT6$^-$ T cells in thymectomized rats. Thus, we have observed that such rats maintain normal ratios (albeit decreasing numbers) of RT6$^-$ and RT6$^+$ T cells for at least an additional 2–6 weeks after thymectomy (Mojcik et al., 1988; see also Results and Table 4); and that diabetes-prone BB rats continue to lack RT6$^+$ T cells at 1 year of age (M. Angelillo, unpublished observation), despite having normal numbers of RT6$^-$ T cells (Greiner et al., 1986), a functional thymus (Angelillo et al., 1988), and a functional RT6 gene (Angelillo et al., 1988).

Because the RT7 alloantigenic marker not only is expressed by T cells in the rat (Lubaroff, 1973; Greiner et al., 1982), but also by B cells (Mojcik et al., 1987) and NK cells (Reynolds et al., 1981), it was important to confirm that the RT7$^+$ RT6$^-$ cells in thymectomized rats were in fact T cells. This was done in three ways. First, we used a titer of anti-RT7.1 mAb that reacts with T cells but not detectably with B cells (Mojcik et al., 1987) to stain the RT6$^-$ lymphocytes (see Table 5). Second, we demonstrated that the OX19 (CD5) pan-T-cell antigen, which is absent from NK cells (Reynolds et

TABLE 7

Number ($\times 10^{-6}$) of RT6$^+$ and RT6$^-$ T Cells and sIg$^+$ B Cells in Spleen of Thymectomized and/or Anti-RT6.1 (DS4.23) mAb-Treated LEW Rats[a]

| Treatment of rats | | T cells | | | B cells |
|---|---|---|---|---|---|
| Tx | DS4.23 | RT7$^+$ | RT6$^+$ | RT6$^-$ | sIg$^+$ |
| − | − | 189.6 ± 25.0 | 98.5 ± 15.0 | 91.1 ± 10.1 | 112.4 ± 12.9 |
| + | − | 111.1 ± 23.6 | 64.3 ± 9.7 | 46.8 ± 14.8 | 99.1 ± 11.7 |
| − | + | 91.2 ± 10.2 | 0.0 ± 0.0 | 91.2 ± 10.2 | 111.5 ± 11.7 |
| + | + | 50.6 ± 6.1 | 0.0 ± 0.0 | 50.6 ± 6.1 | 122.7 ± 17.2 |

[a]See footnote from Table 5 for treatment of rats. The numbers of RT7$^+$ and RT6$^+$ T cells and sIg$^+$ B cells were determined by multiplying the percentage of cells that expressed these antigens by the total number of nucleated spleen cells per animal. The number of RT6$^-$ T cells was calculated by subtracting the number of RT6$^+$ cells from the number of RT7$^+$ cells in the spleen of each animal. Results represent the mean ($\times 10^{-6}$) ± S.D. of three animals per group.

al., 1981; Mason et al., 1983), was expressed by approximately 70% of the $RT6^-$ $RT7^+$ spleen cells (see Table 6). Third, we demonstrated that approximately 70% of the $RT6^-$ $RT7^+$ spleen cells expressed the recently described R73 $\alpha/\beta$ TCR framework antigen (Hunig et al., 1989).

Additional phenotypic analyses indicated that the population of true $RT6^-$ T cells contains both W3/$25^+$ (CD4) and $OX8^+$ (CD8) subsets. The fact that the sum of the percentages of the W3/$25^+$ and $OX8^+$ cells in Table 5 exceeds that of total T cells ($RT7^+$, $R73^+$) probably reflects the expression of W3/25 by macrophages as well as by T cells (Jeffries et al., 1985). Thus, by computing the RT7:OX8 ratio instead of the W3/25:OX8 ratio, it can be seen that the representation of $CD8^+$ cells (and, hence, of $CD4^+$ cells) among the true $RT6^-$ T cells is approximately equal to that among the total population of T cells in normal spleen (2.3:1 vs. 2.6:1) and lymph node (3.1:1 vs 3.1:1).

Although several functional properties have been attributed to $RT6^+$ T cells (see Introduction), almost nothing is known about the functions of $RT6^-$ T cells. Therefore, it is of great interest that the susceptibility of lymphopenic BB/W strain rats to autoimmune insulin-dependent diabetes mellitus has been found to be associated with the congenital absence of $RT6^+$ T cells; and that the resistance of nonlymphopenic BB/W and normal strain rats to diabetes (and thyroiditis) is associated with the presence of $RT6^+$ T cells (Greiner et al., 1986, 1987; Burnstein et al., 1989; McKeever et al., 1990). These latter studies not only have confirmed the presence of immunoregulatory cells within the $RT6^+$ T-cell subset, but have demonstrated that the autoreactive effector T cells (and/or their precursors) reside within the $RT6^-$ T-cell subset. An analogous situation may also exist in mice, in which there is evidence of a homologue of the rat RT6-differentiation antigen (Koch et al., 1990), and in which defective suppressor T-cell activity has been demonstrated in the NOD stain of diabetic mouse (Serreze and Leiter, 1988; Boitard et al., 1989).

Recent studies have provided additional support for the notion that autoreactive T-cell clonotypes and regulatory T cells thereto selectively reside within the $RT6^-$ and $RT6^+$ subsets, respectively. Chen-Woan and Greiner (1991) have observed that most of the $RT6^-$ T cells in normal rats are strongly positive for the OX22 differentiation antigen (CD45R-hi), whereas most of the $RT6^+$ T cells are weakly OX22 positive (CD45R-lo). This correlation may be significant, inasmuch as Powrie and Mason (1990) have demonstrated that purified CD45R-hi T cells induce organ-specific autoimmunity in congenitally athymic rats, and that CD45R-lo T cells appear to inhibit this autoaggressive activity. In addition, Goldschneider et al. (1991) and Chen-Woan and Goldschneider (1991) have described the release of large numbers of potentially autoreactive T cells from the thymus of Cyclosporine A-treated rats. These cells had an "immature" T-cell phenotype and were $RT6^-$.

Hence, both developmental and functional evidence supports the notion that the population of "true" $RT6^-$ T cells is distinct from pre-$RT6^+$ T cells. Indeed, the observation that the defective generation of $RT6^+$ T cells by BB/W rats can be traced to abnormalities in prothymocytes (Angelillo et al., 1988) raises the possibility that separate progenitors may exist for $RT6^+$ and true $RT6^-$ T cells.

Therefore, it must be cautioned that it still is possible that "true" $RT6^-$ T cells are very early precursors of $RT6^+$ cells (i.e., pre-pre-$RT6^+$ cells), but that they require continued thymic influence to proliferate and differentiate into thymus-independent pre-$RT6^+$ cells. An even more likely possibility is that "true" $RT6^-$ T cells represent antigenically naive T cells (including cells that have avoided negative selection in the thymus), whereas $RT6^+$ T cells represent memory T cells. This has been suggested by studies of the properties of the CD45R-hi and CD45R-lo $CD4^+$ T-cell subsets in both the rat (Powrie and Mason, 1988, 1990; Chen-Woan and Greiner, 1991) and the mouse (Lee et al., 1990). It also is consistent with the observations of Hayakawa and Hardy (1989), who used the 3G11 mAb to detect naive ($3G11^+$) and memory ($3G11^-$) subsets of murine $CD4^+$ T cells. These cells not only were thought to have a precursor-progeny relationship, but appeared to express the functions and lymphokine patterns of TH1- and TH2-helper T cells (Mosman and Coffman, 1989), respectively. Moreover, depending on the nature of the antigenic stimulus to which it was exposed, the $CD4^+$ $3G11^-$ T-cell subset could be subdivided by the 6C10 mAb into two phenotypically stable populations ($6C10^-$ and $6C10^+$), each arising from a $6C10^-$ precursor.

It, therefore, will be important to determine if a "switch" between the "true" $TR6^-$ and the $RT6^+$ subsets of $CD4^+$ rat T cells can be induced by appropriate antigenic stimulation; and whether either of these subsets (or subsets thereof) corresponds to the TH1- or TH2-type of helper T cell. It also will be important to determine if the CD45R-hi subset of $RT6^-$ T cells is the repository of autoreactive T cells in normal as well as BB/W rats, and whether such cells are regulated by the CD45R-lo subset of $RT6^+$ T cells. The answer to these questions may provide important insights into the developmental and functional heterogeneity of the T-cell system, and particularly into the pathogenesis of certain forms of organ-specific autoimmune disorders.

### MATERIALS AND METHODS

### Animals

Five-six-week-old rats of the LEW ($RT1^l$, RT6.1, RT7.1), M520 ($RT1^b$, RT6.2, RT7.1), and BUF ($RT1^b$, RT6.1, RT7.2) and WF ($RT1^u$, RT6.2, RT7.2) inbred strains were obtained from the Mammalian Genetics and Animal Production Section of the National Cancer Institute, Frederick, MD. Rats were maintained in laminar-flow hoods on commercial rat chow and acidified chlorinated water (pH 2.2; free chlorine 5–10 ppm) ad libitum.

### Antibodies

Hybridoma cell lines secreting the rat mAb to the postthymic RT6.1 (DS4.23), RT6.2 (6A5), and the pan-T cell RT7.1 (BC84) and RT7.2 (8G6.1) alloantigens are maintained in our laboratory and previously have been characterized (Ely et al., 1983; Milford et al., 1983; Mojcik et al., 1987). Fluorescein conjugation of the DS4.23, 6A5, BC84, and 8G6.1 mAb was done according to the method of The and Feltkamp (1970). Conjugation of biotin to DS4.23 was performed according to the method of Bayer and Wilcheck (1974). Fluorescein (FITC)-conjugated OX19 (CD5) mAb was kindly provided by Dr. M. Chen-Woan, Hartford Hospital, Hartford, CT. An FITC-conjugated mouse mAb to the R73 rat alpha/beta T-cell antigen-receptor framework (Hunig et al., 1989) was purchased from Bioproducts for Science, Inc., Indianapolis, IN. An $F(ab')_2$ fragment of a fluorescein (FITC)-conjugated rabbit antirat IgG (heavy- and light-chain specific) was purchased from Cappell Laboratories, Dowington, PA. Cross-reacting antibodies to mouse Ig were removed by passage over a mouse Ig Sepharose 4B affinity column (Greiner et al., 1987). An affinity-isolated, phycoerythrin (PE)-conjugated goat antirat IgG (heavy- and light-chain specific, mouse absorbed) was purchased from Caltag Laboratories, South San Francisco, CA; and an affinity-isolated, FITC-conjugated goat antimouse IgG (heavy- and light-chain specific, rat serum absorbed) was purchased from Kirkegaard and Perry Laboratories, Gaithersburg, MD.

### Preparation of Cell Suspensions

Spleen, cervical lymph node, and thymus single-cell suspensions were prepared by gently pressing the tissues through a stainless-steel sieve (50 mesh). Bone marrow was obtained by flushing the femur and tibia with cold medium (HEPES buffered RPMI 1640). Red blood cells were removed from spleen and bone-marrow-cell suspensions by hypotonic lysis in 0.168 M $NH_4Cl$. Cell viability was determined by exclusion of 0.1% trypan blue and was >95% in all cases.

### Immunofluorescence Analysis

Direct and indirect immunofluorescence staining was performed as described previously (Mojcik et al., 1987, 1988). Dual immunofluorescence for the DS4.23 (RT6.1) and 8G6.1 (RT7.2) mAbs was performed using FITC-conjugated 8G6.1 and biotin-conjugated DS4.23 developed with TRITC-avidin. Double labeled cells were analyzed by fluorescence microscopy using narrow-band filters for FITC and TRITC. Dual immunofluoresence for the BC84 (RT7.1) and FITC-OX19 or FITC-R73 mAbs was performed using PE-conjugated goat antirat IgG to develop the BC84. In some experiments, the intensity of fluorescence of the FITC-R73 mAb was increased by developing with FITC-conjugated goat antimouse IgG. Cells doubly labeled for FITC and PE were analyzed by flow microfluorimetry (FACS Analyzer, Becton Dickinson and Co., Sunnyvale, CA). Dead cells were excluded electronically, and the fluorescence channels were precalibrated using fluorescent beads. Controls routinely included an irrelevant primary antibody or omission of the primary antibody.

### Separation of $RT6^+$ and $RT6^-$ T Cells *in vitro*

Enriched suspensions of lymph-node T cells were prepared by passage over nylon wool columns, as described (Julius et al., 1973), and were >95% $OX19^+$ ($CD5^+$) and <5% $sIg^+$ in all cases. The T cells were then labeled with DS4.23 anti-RT6.1 and separated into $RT6^+$ and $RT6^-$ subsets by "panning"

(Wysocki and Sato, 1978). For this purpose, $10 \times 10^6$ labeled cells in 3 ml cold RPMI-1640 medium containing 5% gamma-globulin-free horse serum (GIBCO, Chagrin Falls, OH) were placed in plastic Petri dishes (#1001, Falcon, Oxnard, CA) that had been coated by incubation for 24 hr at 4°C with 10 $\mu l$ of goat antirat IgG in 5 ml PBS. The cells were allowed to settle in the dishes for 30 min at 4°C, the nonadherent ($RT6^-$) T cells were recovered by washing in medium with gentle swirling, and the adherent ($RT6^+$) T cells were recovered by gently scraping the plate with a rubber policman after residual nonadherent cells had been removed by vigorous swirling of medium. The purity of the $RT6^-$ and $RT6^+$ T-cell subsets obtained by this procedure exceeded 95% as determined by immunofluorescence. Only $F(ab')_2$ fragments of anti-RT6.1 and antirat Ig were used so as to prevent opsonization of the positively selected $RT6.1^+$ lymphocytes in adoptive transfer studies (Greiner et al., 1982, 1987).

### Depletion of $RT6^+$ T cells *in vivo*

In some experiments, intact (as opposed to $F(ab')_2$ DS4.23 anti-RT6.1 mAb was used to deplete preexisting $RT6.1^+$ T cells *in vivo*. This mAb has previously been found to specifically eliminate $RT6^+$ T cells, rather than to simply "mask" or modulate the RT6 antigen from the cell surface (Greiner et al., 1987; Woda et al., 1988). This was confirmed by preliminary dose-response experiments in 100–400-g LEW ($RT6.1^+$) rats. The results showed that injection of optimal doses of DS4.23 mAb not only completely depleted $RT6^+$ cells in spleen and lymph node, but proportionately decreased the total number of T cells ($RT7.1^+$) as well (eg see Table 7). As anticipated, the number of B cells was unaffected. Moreover, in control experiments, injection of DS4.23 mAb into WF ($RT6.2^+$) rats, or injection of 6A5 (anti-RT6.2) mAb into LEW ($RT6.1^+$) rats, did not significantly alter the proportion or number of splenic $RT6^+$ T cells detected, thereby demonstrating the alleleic specificity of these mAbs (data not shown).

Based on these and other results (Greiner et al., 1987), a standard dose of 0.3 mg/kg body weight of DS4.23 mAb injected *i.v.* was used to deplete $RT6^+$ T cells in LEW rats. Enrichment of the unaffected $RT6^-$ T cells from these animals was achieved by passage over nylon wool columns, as before.

### Thymectomy

Thymectomy and sham-thymectomy was performed on 4–6-week-old rats under open ether-induced anesthesia as described (Greiner et al., 1982). The completeness of thymectomy was confirmed by gross and histological examination at autopsy.

### Adoptive Transfer Systems

Details of the *i.v.* quantitative adoptive transfer system for prothymocytes have been described previously (Greiner et al., 1984; Goldschneider et al., 1986). Briefly, $25 \times 10^6$ bone marrow cells were injected *i.v.* via the tail vein into irradiated (750 rad) histocompatible, RT6 and/or RT7 alloantigen-disparate recipients. The percentages of donor- and host-origin thymocytes and T cells in the recipients were quantified at various times after reconstitution by flow microfluorometric analysis using the appropriate anti-RT6 and anti-RT7 mAbs.

For adoptive transfer experiments designed to study the developmental potentials of purified $RT6^+$ and $RT6^-$ T cells, the recipient rats were thymectomized, irradiated (750 rad) 4 weeks later, and injected *i.v.* with $5 \times 10^6$ syngeneic bone marrow cells (TXBM rats). Four weeks thereafter, bone marrow cells or lymph-node T cells obtained from histocompatible, RT6- and RT7-disparate donors were injected *i.v.* into these recipients and the appearance of donor-origin $RT6^+$ and/or $RT6^-$ T cells was observed at timed intervals by dual-immunofluorescence analysis for the appropriate RT6 and/or RT7 alloantigens.

### ACKNOWLEDGMENTS

We thank Marianne Angelillo, Anita Wayne, Frances Tausche, and Jerome Murphy for their expert technical assistance, and Ruth Conrod and Rinette Pelletier for their excellent secretarial assistance.

This work was supported by Grants GM-38306 and DK 36024 from the National Institutes of Health, USPHS.

(*Received November* 12, 1990)

(*Accepted November* 19, 1990)

### REFERENCES

Angelillo M., Greiner DL., Mordes J.P., Handler E.S., Nakamura N., McKeever U., and Rossini A.A. (1988). Absence of $RT6^+$ T cells in diabetes-prone Biobreeding/Worcester rats is due to genetic and cell development defects. J. Immunol. **141**: 4146–4151.

Bayer E., and Wilcheck M. (1974). Insolubilized biotin for the purification of avidin. *In:* Methods in Enzymology, Jakoby

W.B., and Wilcheck M., Eds. (New York: Academic Press), pp. 265–267.

Boitard C., Yasunami R., and Bach J.F. (1989). T cell-mediated inhibition of the transfer of autoimmune diabetes in NOD mice. J. Exp. Med. **169:** 1669–1680.

Burnstein D., Mordes J.P., Greiner D.L., Stein D., Nakamura N., Handler E.S., and Rossini A.A. (1989). Prevention of diabetes in BB/Wor rat by single transfusion of spleen cells. Diabetes **38:** 24–30.

Chen-Woan M., and Goldschneider I. (1991). Cyclosporin A treatment causes the appearance in rat lymph node of T cells having the antigenic phenotypes of cortical thymocytes. Transplantation **51.**

Chen-Woan M., and Greiner D.L. (1991). Mechanisms of allograft prolongation in RT6 T cell-depleted rats. Transplant Proc. **23:** 143–146.

Ely J.M., Grenier D.L., Lubaroff D.M., and Fitch F.W. (1983). Characterization of monoclonal antibodies which define rat T cell alloantigens. J. Immunol. **130:** 2798–2803.

Ernst D.N., and Lubaroff D.M. (1984). Membrane antigen phenotype of sensitized T lymphocytes mediating tuberculin-delayed hypersensitivity in rats. Cell. Immunol. **88:** 436–452.

Everett N.B., Caffrey, R.W., and Rieke W.O. (1964). Recirculation of lymphocytes. Annu. N.Y. Acad. Sci. **113:** 887–897.

Goldschneider I., Hosseinzedah H., and Chen-Woan M. (1991). Short-term treatment of rats with Cyclosporin A causes the appearance of lymph node T cells that resemble cortical thymocytes. Transplant Proc. **23:** 122–125.

Goldschneider I., Komschlies K., and Greiner D.L. (1986). Studies of thymocytopoiesis in rats and mice. I. Kinetics of appearance of thymocytes using a direct intrathymic (i.t.) adoptive transfer assay for thymocyte precursors. J. Exp. Med. **163:** 1–17.

Greiner D.L., Goldschneider I., and Barton R.W. (1982). Identification of thymocyte progenitors in hemopoietic tissues of the rat. II. Enrichment of functional prothymocytes on the fluorescence-activated cell sorter. J. Exp. Med. **156:** 1448–1460.

Greiner D.L., Goldschneider I., and Lubaroff D.M. (1984). Identification of thymocyte progenitors in hemopoietic tissues of the rat. I. A quantitative assay system for thymocytes regeneration. Thymus **6:** 181–199.

Greiner D.L., Handler E.S., Nakano K., Mordes J.P., and Rossini A.A. (1986). Absence of the RT6 T cell subset in diabetes-prone BB/W rats. J. Immunol. **136:** 148–151.

Greiner D.L., Mordes J.P., Handler E.S., Angelillo M., Nakamura N., and Rossini A.A. (1987). Depletion of RT6.1⁺ T lymphocytes induces diabetes in resistant Biobreeding/Worcester (BB/W) rats. J. Exp. Med. **166:** 461–475.

Greiner D.L., Reynolds C.W., and Lubaroff D.M. (1982). Maturation of functional T lymphocyte subpopulations in the rat. Thymus **4:** 77–90.

Greiner D.L., Rossini A.A., Handler E.S., Mordes J.P., and Nakano K. (1986). Absence of the RT-6 T cell subset in diabetes-prone BB/W rats. J. Immunol. **136:** 148–151.

Hayakawa K., and Hardy R.R. (1989). Phenotypic and functional alteration of CD4⁺ T cells after antigenic stimulation. Resolution of two populations of memory T cells that both secrete interleukin 4. J. Exp. Med. **169:** 2245–2250.

Hunig T., Wallny H.-J., Hartley J.K., Lawetzky A., and Tiefenthaler G. (1989). A monoclonal antibody to a constant determinant of the rat T cell antigen receptor that induces T cell activation. J. Exp. Med. **169:** 73–86.

Hunt H.D., and Lubaroff D.M. (1985). Interaction of T lymphocyte subpopulations bearing the RT6 alloantigen. Transplant Proc. **17:** 1861–1863.

Hunt H.D., and Lubaroff D.M. (1987). Changes in membrane antigen phenotype of T cells during lectin activation. Transplant Proc. **19:** 3179–3180.

Jeffries W.A., Green J.R., and Williams A.F. (1985). Authentic T helper CD4 (W3/25) antigen on rat peritoneal macrophages. J. Exp. Med. **162:** 117–127.

Julius M.H., Simpson E., and Herzenberg L.A. (1973). A rapid method for the isolation of functional thymus-derived murine lymphocytes. Eur. J. Immunol. **3:** 645–649.

Koch F., Haag F., and Thiele H.G. (1990). Nucleotide and deduced amino acid sequence of the BALB/c mouse homologue of the rat T cell differentiation marker RT6. *Nucl. Acids Res.* **18:** 3636.

Koch F., Kashan A., and Thiele H.-G. (1988). The rat T cell differentiation marker RT6.1 is more polymorphic than its alloantigenic counterpart RT6.2. J. Immunol. **65:** 259–265.

Koch F., Thiele H.-G., and Low M.C. (1986). Release of the rat T cell alloantigen RT6.2 from cell membranes by phosphotidylinositol-specific phospholipase C. J. Exp. Med. **164:** 1338–1343.

Lee W.T., Yin X.-M., and Vitetta E.S. (1990). Functional ontogenetic analysis of murine CD45R$^{hi}$ and CD45R$^{lo}$ CD4⁺ T cells. J. Immunol. **144:** 3288–3295.

Lubaroff D.M. (1973). An alloantigenic marker on rat thymus and thymus-derived cells. Transplant Proc. **5:** 115–118.

Lubaroff D.M., Greiner D.L., and Reynolds C.W. (1979). Investigation of T-lymphocyte subpopulations in the rat using alloantigenic markers. Transplant Proc. **11:** 1092–1094.

Mason D.W., Authur R.P., Dallman M.J., Green J.R., Spickett G.P., and Thomas M.L. (1983). Functions of rat T-lymphocyte subsets isolated by means of monoclonal antibodies. Immunol. Rev. **74:** 57–82.

McKeever U., Mordes J.P., Greiner D.L., Appel M.C., Rozing J., Handler E.S., and Rossini A.A. (1990). Adoptive transfer of autoimmune diabetes and thyroiditis to athymic rats. Proc. Nat. Acad. Sci. USA. **87:** 7618–7622.

Milford E.L., Paradysc J.M., and Carpenter C.B. (1983). A monoclonal antibody that detects HLA-B7-associated polymorphism. Transplant Proc. **15:** 1974–1975.

Mojcik C.F., Greiner D.L., Goldschneider I., and Lubaroff D.M. (1987). Monoclonal antibodies to RT7 and LCA antigens in the rat: Cell distribution and segregation analysis. Hybridoma **6:** 531–543.

Mojcik C.F., Greiner D.L., Medlock E.S., Komschlies K.L., and Goldschneider I. (1988). Characterization of RT6 bearing rat lymphocytes. I. Ontogeny of the RT6 subset. Cell. Immunol. **114:** 336–346.

Mosmann T.R., and Coffman R.L. (1989). TH1 and TH2 cells: Different patterns of lymphokine secretion lead to different functional properties. Annu. Rev. Immunol. **7:** 145–173.

Powrie F., and Mason D.W. (1988). Phenotypic and functional heterogeneity of CD4⁺ T cells. Immunol. Today **9:** 274–277.

Powrie F., and Mason D.W. (1990). OX-22$^{high}$ CD4⁺ T cells induce wasting disease with mutliple organ pathology; Prevention by the OX-22$^{low}$ subset. J. Exp. Med. **172:** 1701–1708.

Reynolds, C.W., Sharrow S.O., Ortaldo J.R., and Herberman R.B. (1981). Natural killer activity in the rat. II. Analysis of surface antigens on LGL by flow cytometry. J. Immunol. **127:** 2204–2208.

Rossini A.A., Mordes J.P., and Greiner D.L. (1987). The pathologenesis of autoimmune diabetes mellitus. Cur. Top. Immunol. **2:** 598–603.

Rossini A.A., Mordes J.P., Greiner D.L., Nakano K., Like A.A., and Handler E.S. (1986). Spleen cell transfusion in the Bio-Breeding/Worcester rat: Prevention of diabetes, major histocompatibility complex restriction and long term persistence of transfused cells. J. Clin. Invest. **77:** 1399–1401.

Serreze D.V., and Leiter E.H. (1988). Defective activation of T suppressor cell function in nonobese diabetic mice. Potential relation to cytokine deficiencies. J. Immunol. **140:** 3801–3807.

The T.H., and Feltkamp T.E.W. (1970). Conjugation of fluorescein isothiocynate to antibodies. I. Experiments on the condition of conjugation. J. Immunol. **18:** 865–873.

Thiele H.-G., Koch F., and Kashan A. (1987). Postnatal distribution profiles of Thy1⁺ and RT6⁺ cells in peripheral lymph nodes of DA rats. Transplant Proc. **19:** 3157–3160.

Woda B.A., Padden C., and McFadden M.L. (1988). T helper ($T_H$) cells in the Biobreeding Worcester (BB/Wor) rat are subdivided into distinct subpopulations by the OX22 and RT6 antibodies. Diabetes **37:** 788.

Wysocki L.J., and Sato V.L. (1978). "Panning" for lymphocytes: A method for cell selection. Proc. Natl. Acad. Sci. USA **75:** 2844–2848.

